# Single-Cell Proteomics: The Critical Role of Nanotechnology

**DOI:** 10.3390/ijms23126707

**Published:** 2022-06-16

**Authors:** Carlota Arias-Hidalgo, Pablo Juanes-Velasco, Alicia Landeira-Viñuela, Marina L. García-Vaquero, Enrique Montalvillo, Rafael Góngora, Ángela-Patricia Hernández, Manuel Fuentes

**Affiliations:** 1 Department of Medicine and General Cytometry Service-Nucleus, CIBERONC, Cancer Research Centre (IBMCC/CSIC/USAL/IBSAL), 37007 Salamanca, Spain; carlotaarias2009@gmail.com (C.A.-H.); pablojuanesvelasco@usal.es (P.J.-V.); alavi29@usal.es (A.L.-V.); marina.luquegv@gmail.com (M.L.G.-V.); emontalvillo@usal.es (E.M.); rgongora@usal.es (R.G.); 2 Department of Pharmaceutical Sciences: Organic Chemistry, Faculty of Pharmacy, University of Salamanca, CIETUS, IBSAL, 37007 Salamanca, Spain; 3 Proteomics Unit, Cancer Research Centre (IBMCC/CSIC/USAL/IBSAL), 37007 Salamanca, Spain

**Keywords:** single-cell proteomics, nanotechnology, mass-spectrometry, antibodies, biological variability, cancer immunotherapy, clinical research

## Abstract

In single-cell analysis, biological variability can be attributed to individual cells, their specific state, and the ability to respond to external stimuli, which are determined by protein abundance and their relative alterations. Mass spectrometry (MS)-based proteomics (e.g., SCoPE-MS and SCoPE2) can be used as a non-targeted method to detect molecules across hundreds of individual cells. To achieve high-throughput investigation, novel approaches in Single-Cell Proteomics (SCP) are needed to identify and quantify proteins as accurately as possible. Controlling sample preparation prior to LC-MS analysis is critical, as it influences sensitivity, robustness, and reproducibility. Several nanotechnological approaches have been developed for the removal of cellular debris, salts, and detergents, and to facilitate systematic sample processing at the nano- and microfluidic scale. In addition, nanotechnology has enabled high-throughput proteomics analysis, which have required the improvement of software tools, such as DART-ID or DO-MS, which are also fundamental for addressing key biological questions. Single-cell proteomics has many applications in nanomedicine and biomedical research, including advanced cancer immunotherapies or biomarker characterization, among others; and novel methods allow the quantification of more than a thousand proteins while analyzing hundreds of single cells.

## 1. Introduction

Complex biological processes are based on dynamic interactions between individual cells, involving in many cases multiple cell types as well as different states and susceptibilities [1]. Traditional bulk tissue analysis averages all the differences between cell diversity presented in most of the biological/biomedical samples, whereas single-cell analysis allows the characterization of each individual cell, studying—at the single cell level—its genomics, transcriptomics, proteomics, metabolomics, and cell–cell interactions. This analysis enables the discovery and classification of unknown cell states [2].

**Figure 1:**
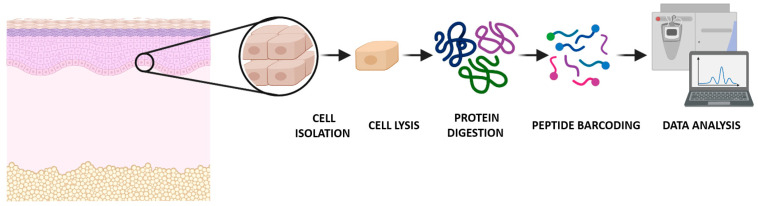
Global overview of the single-cell proteomics workflow analyzed by mass spectrometry.

Most single-cell studies are focused on nucleic acids, especially the genes expressed at the cellular level [3]. However, the nucleic acid-based technologies do not take into account an important group of biological regulators in the cell: the proteins [2]. Proteins are the workhorses of the cell, impacting all aspects of cellular processes in all physiological situations. At the single-cell level, nucleic acids behave in a predictable way, but the proteome has a wide range of different chemistries, interactions, dynamics, and abundances [3]. Its acute state (i.e., the proteotype) depends on both the genotype and external perturbations and/or stimuli. Therefore, quantitative analysis of the proteome dynamics, including post-translational modifications (PTMs) and their connection to phenotypes and diseases, has become indispensable in biological and clinical research. Since there is a lack of an equivalent—at the protein level—to DNA amplification by PCR, any protein detection technique must be sensitive enough to identify them, even at wide dynamic range of the protein concentration in the cell [3,4]. Proteomics aims to identify, characterize, and quantify all the protein isoforms in a cell, tissue, organ, or organism of interest [3]. Global proteome measurements based on mass spectrometry (MS) and/or tandem mass spectrometry (MS/MS), which is used to improve the specificity of the mass spectrometer coupling two analyzers by using a collision cell) have been performed with biological samples that comprise thousands or millions of cells. This provides a quantitative protein expression profile but does not account for heterogeneity within the sample [1]. Novel nanoscale MS approaches—that identify and quantify proteins in a more deep and accurate manner—are promising tools in the development of single-cell protein analysis. These proteomics technologies will facilitate high-throughput investigation of fundamental biological questions, such as protein-binding signaling mechanisms or protein modifications [2] (Figure 1).

Currently, high-content data sets of single-cell genomic and transcriptomic data can be generated and, as Single-Cell Proteomics (SCP) emerges, researchers will be able to integrate single-cell mRNA and proteomic measurements [5]. The abundance and role of many proteins are regulated by PTMs and degradation that cannot be inferred from genomic and transcriptomic approaches, making proteomics essential for determine protein patterns relevant to disease diagnosis and/or drugs response, among others. Furthermore, genomic and transcriptomic sequencing cannot directly explain protein localization and protein–protein interactions, which are critical for numerous signaling pathways [2,3,6,7]. The protein abundance in a cell can vary between isogenic single cells, which affects regulatory roles and controls cell fate during apoptosis and cell proliferation. Early investigations of cellular heterogeneity were focused on isogenic bacterial populations growing in the same culture, demonstrating that individual bacteria varied in terms of persistence, λ phage burst size, β-galactosidase production, and chemotactic behavior [8,9]. Measurements using GFP revealed unexpected variability in protein levels expressed from the same promoter, which was interpreted as biochemical noise comprising intrinsic (from the biochemical process of transcription and translation) and extrinsic (external environmental fluctuations) components [10,11]. In many cases, protein abundance variability reflects different cellular states that can lead to a wide diversity of functional outcomes, while other experiments have demonstrated that gene expression heterogeneity can be used to respond to environmental changes in a dynamic manner [11,12,13].

Several human health situations are related to disturbances of the immune system (autoimmune diseases, infectious diseases, or chronic inflammation) and other pathologies with different ontogeny (i.e., cancer, neurodegenerative disorders) [14]. Recently, it was described that approximately 20% of cancers (solid tumors and onco-hematological pathologies) can be caused by infectious agents, with *Helicobacter pylori*, hepatitis C virus, or Kaposi’s sarcoma-associated herpesvirus being some examples [15,16]. Novel therapeutic approaches for the treatment of tumors have emerged as a consequence of the close relationship between the immune system and the different diseases. Among these are vaccination, monoclonal antibodies, immune checkpoint inhibitors, adoptive T-cell transfer, and oncolytic virus therapy [17]. The understanding of cellular and molecular mechanisms involved in cancer makes it possible to identify potential targets—for novel onco-immunotherapies—based on the modulation and regulatory control of immune response [18]. Tumor genomes are disrupted at numerous sites, by either point mutations or more apparent alterations, such as chromosomal complement changes. Thus, cancer cells have defects in signaling pathways that regulate normal cell proliferation and homeostasis, and different tumors have a wide variety of genotypes [17]. In these heterogeneous cell populations, a wide variety of proteins acting together control cellular decisions. Characterizing these complex systems demands measurements of thousands of proteins through thousands of single cells. Consequently, novel methods for single-cell protein analysis need to be developed, which, combined with transcriptome and metabolome analysis of single cells, will help provide crucial data for the development of quantitative systems biology [11]. Moreover, due to this complexity and diversity, the behavior of cancer cells in response to drugs is also heterogeneous, requiring proteome analysis at the single-cell level. A study has demonstrated that SCP techniques are now quantitative enough to address the effects of drugs on target proteins, thereby leading to single-cell chemical proteomics [19]. Therefore, through the use of nanoparticles and/or nanostructured surfaces, it will help reveal mechanisms that lie behind health and disease, as the characterization of cell–cell communication, cell microenvironment and migration, immune suppression, or cell death, among many others, are critical in the development of treatments for multiple pathologies [2,3].

## 2. Sample Preparation

Single-cell heterogeneity is gaining relevance due to recent advances in single-cell RNA and protein analysis, characterizing processes such as the cell division cycle [20]. Traditionally, fluorescent-labeled proteins and affinity reagents have been used in SCP analyses; however, nanoscale MS analyses could increase the specificity and coverage of protein quantification, since they are able to analyze thousands of proteins within individual cells [20]. One of the most common workflows to characterize complex proteomes is based on a bottom-up MS approach, which aims to quantitatively extract and digest proteins chemically and enzymatically from isolated cells or tissues, generate peptides and deliver them to a ready-to-analyze separation platform in sufficient quantities to enable a robust measurement, such as liquid chromatography coupled to a mass spectrometer via an electrospray interface [1,21].

The sample preparation preceding MS analysis is a critical step, as it greatly impacts sensitivity, robustness, and reproducibility [21]. Sample preparation has typically been performed following non-automated preparation protocols, which may be highly influenced by operator skill, affecting the preparation consistency and leading to unreliable results. Hence, sample preparation methods are needed to reduce user interactions to a minimum and make preparation steps more robust. Additionally, bulk samples are often prepared using relatively large volumes and chemicals (detergents or chaotropic agents like urea) that are incompatible with quantitative liquid chromatography and tandem mass spectrometry (LC-MS/MS) analysis and require removal by clean-up procedures [20,22]. Several nanotechnological strategies have been developed in the last few years to remove salts, detergents, and cellular debris, as well as to simplify sample manipulations in a single vessel. Among these methods are affinity-based assays, electrophoretic approaches, membrane filtration, and protein precipitation [1,21]; for example, filter-aided sample preparation (FASP), in-StageTip digestion (iST) and single-pot solid-phase enhanced sample preparation (SP3) have become increasingly popular due to their optimizing the sample amount and maximizing analyte recovery [21] (Table 1). FASP enables most contaminants (including salts and lipids) to be removed by centrifugation through an ultrafiltration device with molecular weight cut-off. Proteins are trapped on a filter membrane, where they are enzymatically digested in peptides small enough to pass through the filter and be collected by centrifugation. It has been widely adapted for different applications, including the characterization of different cell and tissue types, large-scale ubiqitinome screening, N-glycoproteome mapping, or brain phosphoproteome analysis [21]. SP3 and iST are both “single-vessel” approaches that minimize the loss of sample by making the workflow simpler. On one hand, SP3 is performed in a single container with paramagnetic beads functionalized at the surface—for example, carboxylate-coated—to capture proteins in hydrophilic layers, followed by the immobilization of the beads inside a magnetic field. Contaminants, including chaotropes and detergents, can then be removed by organic solvent wash (i.e., ethanol and acetonitrile) [21]. This technique has been successfully applied in different studies, including a proteomic quantitative analysis of the tissue substructures of mouse kidney [23], and human oocytes [21,24]. On the other hand, in iST, the cell lysis and further protein processing and digestion are performed in stop-and-go extraction tips (StageTips) with a C18 disk inserted. This method allows full sample preparation in a unique reactor, taking advantage of a FASP-like reaction vessel that avoids the filtration step, yet iST is unable to remove some chemical reagents, such as SDS. It also facilitates the final clean-up of the peptide by solid phase extraction (SPE) [21]. Both SP3 and iST methods have been demonstrated to offer high proteome coverage, reproducibility, and accuracy, even when only 1 μg of protein is handled. Furthermore, aggressive sample cleanup and protein extraction steps required for bulk workflows may not be necessary for samples with low cell input, simplifying the preparation protocol [1]. However, losses are more significant with low-abundance samples, and cleanup steps make the automation complex, since it introduces variability between samples. By avoiding these steps, losses can be reduced while increasing throughput and consistency [20,21,22].

Focused Acoustic Sonication (FAS) is a cell lysis method with no MS-incompatible chemicals and, therefore, can be used in LC-MS/MS without further cleaning. However, while FAS allows a clean lysis, it requires large volumes (5–10 µL), resulting in low throughput, which limits its potential for SCP [22]. To relieve these limitations, an inexpensive and easily automated method for lysing cells was developed, allowing a high-throughput format and small lysis volumes. Minimal ProteOmic sample Preparation (mPOP) performs the lysis of culture-grown mammalian cells by a freeze–heat cycle in pure water droplets. This allows sample preparation using a digestion buffer compatible with MS (Triethylammonium bicarbonate, pH 8.0), thus eliminating cleanup steps and allowing easy self-coupling of mPOP sample preparation with PCR thermocyclers, further reducing volumes from 10 to 1 µL and allowing sample preparation in 96/384-well plates for simultaneous multi-sample processing, increasing the throughput over 100-fold (Table 1). Bulk samples processed by both mPOP and urea-based methods showed that mPOP achieves complete cell lysis and accurate quantification of proteins from the nucleus, cytosol, mitochondrion, and cell membrane. The results demonstrated that mPOP increases the efficiency of proteome extraction, the accuracy of quantification and the depth of proteome coverage [22].

**Figure 2:**
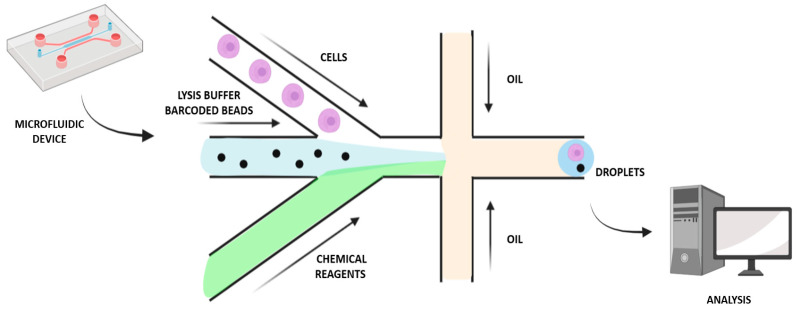
Droplet-based microfluidic approach for single-cell analysis and isolation.

To make further progress in single-cell MS proteomics, a miniaturized and massively parallel sample preparation method was developed for high-throughput characterization: automated nano-ProteOmic sample Preparation (nPOP) (Table 1). This novel procedure makes it possible to increase the experimental batch size while also reducing batch effects, since it relies on piezo acoustic dispensing to isolate cells individually in volumes lower than 20 nL and performs all steps of the protocol in small droplets on a fluorocarbon-coated slide. The workflow sample preparation includes cell isolation and lysis and protein digestion, followed by labeling of peptides and clustering of samples [20]. To assess the nPOP performance for single-cell analysis, two cell lines, Hela cells and U-937 monocytes, were used, sorting cells by both type and cycle phase. This allowed a reduction of sample volume by 100-fold compared to the mPOP method, enabling a more comprehensive investigation of the cell division cycle. Furthermore, characterization of cells according to their division cycle phase was possible by integrating nPOP with the Single-Cell ProtEomics by Mass Spectrometry (SCoPE2) workflow. Analysis of the proteins in the cell lines U-937 and HeLa revealed significant similarities and distinctions during cell cycle progression; hence, nPOP allowed a deeper single-cell proteomic analysis than the mPOP sample preparation method [20]. In addition, microfluidics-based toolkits for SCP are also emerging, offering the following benefits: i. More than twenty functional proteins can be simultaneously analyzed based on individual cell statistical numbers; ii. Cell behaviors (e.g., motility) and protein assays can be correlated; iii. Measurements of cell–cell interactions can be performed by the extensions to quantified cell populations; iv. To allow further analysis and culturing, rare cells can be identified and functionally separated; v. Some assays can provide a conduit between biology and the physicochemical laws [25]. Using open microfluidic platforms will reduce the dimensions and maintain the general form factor of the microwell plate, thereby minimizing surface exposure and enabling sample recovery through nano-pipetting [1,25]. Other approaches have been developed by contact pin-printing methods in which pipetting robots automatically dispense liquids at the Transmission Electron Microscopy grid, such as a capillary-driven microfluidic single-use device [26]. These approaches have the advantages of reducing the volumes of the sample as well as providing automation possibilities, while they also have some disadvantages, such as the need for special instrumentation, and representing a significant increase in complexity and being more time consuming than the manual protocol [26]. Mukhitov et al. suggested a microfluidic device for the preparation of the grids, in which the grid is contained in a microfluidic canal and the liquid for sample preparation is driven by an external pressure pump, thereby improving preparation consistency [27]. Moreover, flow cytometry miniaturization and blood cell counting have been attracting attention in recent years [28]. Reducing the volumes for sample preparation can be beneficial for minimizing adsorption losses and increasing sample concentrations, so sample reaction with trypsin and other reagents can be more efficient [1]. Automated sample handling is necessary in order to standardize the sample preparation workflow, the operations of which are typically performed manually. A fluid standardized plumbing system in which the device allows the full volume to be mixed and processed could enable large volumes to be processed, leading to reduced costs, and increased diagnostic accuracy [28]. For example, a droplet-based microfluidic approach enables the miniaturization of reactions possible by segmentation into droplets containing femto- to micro-liter volumes, which assists biochemical screening and enzyme kinetic studies and assays (Figure 2). As each compartmented droplet performs a single reaction, several reactions can be carried out in parallel. Digital microfluidics, a droplet manipulation method using electrowetting, enables sequential operations to be performed on slide-fixed cell and tissue samples using low volumes in a non-continuous manner. The ideal protocol would automate techniques for cell staining on a microfluidic device for efficient and uniform labeling using immunocytochemical stains and improving the yield of cytogenetic analysis [28].

Given the lack of amplification methods, the wide variety of species, the relatively low abundance, and the large dynamic range of proteins, they have barely been studied in single-cell research compared to other macromolecules [29]. Therefore, sensitive separation techniques are required in SCP. Capillary electrophoresis has been extensively used in complex biological sample separation and analysis due to its fast analysis speed, low cost, and high separation efficiency. Its distinctive capability lies in the extraction and transfer of cellular or subcellular region components using capillaries smaller than a cell’s size. Using this method also provides less substrate interference and minimal oxidative damage to the cells. However, it is mainly focused on large cell research due to the significant sample loss, unstable interface, and issues of reproducibility [29,30]. In addition, ultrathin-layer gel electrophoresis has also been developed. It combines the advantages of conventional slab-gel electrophoresis (multilane format) and capillary gel electrophoresis, allowing automatic, fast, high-throughput separations over a wide range of molecular weights. The scale down of the process enables better yields due to the possibility of higher voltages, thus speeding up the analysis. In fact, miniaturization results in faster, easier, less expensive, and more convenient analyses, achieving massively parallel assays [31,32]. Nevertheless, liquid chromatography, especially nanoLC, is used more extensively in SCP research. It relies mainly on its reproducibility, nanoliter injection volume, low flow rate, reduced sample loss and easy compatibility with mass spectrometry. More than 1000 proteins have been detected in isolated HeLa cells using this approach [29]. However, the suitability of cell lysis, the protein pre-treatment integrity and efficiency, and peptide labeling are major factors affecting the overall number and types of protein identification [29,30]. Multidimensional LC analysis is a sample separation process with at least, and most typically, two different chromatographic separation columns or dimensions. The principal advantage is the dramatic increase in peak capacity, known as the “product rule” [33]. As a result, it allows the separation of difficult-to-resolve components, or samples with a high number of constituents. The broad and complex composition of biological samples requires multidimensional methodologies to sufficiently separate components prior to MS characterization [33].

## 3. Technological Development

Biological variations can be attributed—in single-cell analysis—to cells individually, instead of being averaged from a complex tissue [34]. Currently, the technology is mainly limited to imaging and deep sequencing, with relatively limited capabilities, but novel approaches would help resolve the heterogeneity within the cell resolution [1,2]. Single-cell RNA-seq methods can measure transcriptomes from individual cells, allowing the classification of cell populations, often uncovering undetected but biologically relevant subpopulations. Still, they can only reflect a fraction of the transcriptional gene expression profile and mRNA levels are insufficient to fully characterize, comprehend and monitor biological systems. It cannot capture PTMs or explain proteins changes across human tissues since the differences implicate regulatory mechanism based on tissue-specific proteins [11]. SCP techniques are able to quantify phosphorylation and other modifications by single-cell mass cytometry, single-cell Western blots, and immunoassays, among others. These have the potential to open new possibilities to explore the dynamics of phosphorylation and monitor cells as they evolve due to mutations [5].

**Figure 3:**
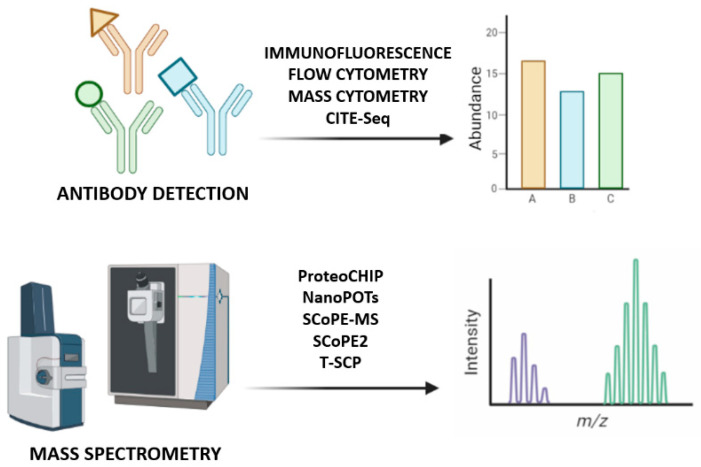
Single-cell proteomics identification methods. At the top of the image, antibodies bind to the epitopes of their proteins and are detected to quantify the corresponding protein; at the bottom, mass spectrometry approaches introduce a non-targeted analysis to identify all proteins based on their mass-to-charge ratio.

Some proteomics methods rely on antibodies to detect chosen targets in single cells [2,34]. However, antibody-based techniques such as immunofluorescence, flow and mass cytometry or cellular indexing of transcriptomes and epitopes by sequencing, have limited specificity and are able to measure a small number of proteins previously selected, while high-resolution microscopy provides single-cell measurements [1]. Nevertheless, not all proteins have their respective antibodies, some of them have low specificity for their targets and bind to proteins and/or their PTMs weakly or non-specifically [3], and have low multiplexing capacities [35]. Although some highly specific and well-validated antibodies can be useful for analyzing many cells, they target specific proteins previously known, limiting the studies to that portion of the proteome [2]. MS-based proteomics can be used instead, since it consists in a non-targeted method to identify and quantify molecules based on their mass and charge, measuring non-modified proteins and PTMs within its range of detection, for example, by chemical-labeling approaches that introduce hundreds of cells into the MS. However, to answer biological questions, it is imperative to raise the sensitivity, robustness, and quantitative accuracy, challenging the understanding of protein interactions and functions at single-cell resolution [2,3,34,36] (Figure 3).

A high impact on the biomedical field is expected as soon as an in-depth characterization and an unbiased protein expression profile of individual cells is accomplished, thereby unravelling microenvironmental factors that either enhance or inhibit tumor growth, identifying previously unknown cellular subpopulations, or developing pathways that may be missed in bulk measurements [1,34]. Novel methodologies for SCP are thus needed to analyze a wide variety of membrane-bound, intracellular, and extracellular proteins at single-cell level. In addition, it further enables accessibility of multiple limited samples such as rare circulating tumor cells or fine needle aspiration biopsies, mapping protein expression with high spatial resolution across tissues [1,2,36]. Some labs are considering ways related to cytometry to collect single-cell data, using single-cell mass cytometry during hematopoiesis to capture cell-fate decisions, and tracking how transcription factors expression change [5]. Recent progress in sample handling, separations and instrumentation has enabled over 1000 proteins to be quantified from single mammalian cells [1]. A new multiplexed single-cell MS-based proteomics device called proteoCHIP mapped over 2000 proteins across 158 cells from two different human cell types [3]. Another novel design is called NanoPOTS [37], a nanoliter-scale microtiter plate in which each hydrophobic well has a small hydrophilic ‘pedestal’ on which cells are deposited and prepared. If combined with ultrasensitive liquid chromatography-MS, NanoPOTS also allows the identification of 1500 to 3000 proteins within a range from 10 to 140 single cells, respectively [37], and has demonstrated promising results in decreasing sample loss to a minimum [35].

As stated above, mass spectrometry has become a central technology in the field of proteomics, although further understanding of biological components by determining their distribution is needed. This anatomical dimension has been added by mass spectrometry imaging, especially by MALDI-MSI [38]. It offers the possibility of targeting biomarkers in a variety of diseases, bringing these technologies in line with the goals of clinical proteomics, including early disease detection, selection of therapeutic combinations based on the patient’s disease-specific protein network, and rational redirection of therapy based on changes in the diseased protein network associated with drug resistance, among others [38]. MALDI is the use of a matrix-assisted laser for desorption ionization of the sample that enables the correlation of molecular information with histology by preserving the spatial localization of analytes after MS measurement. It is a label-free approach and allows multiplex analysis of hundreds to thousands of molecules in the same tissue section simultaneously, which brings a new quality of molecular data and tissue research [39].

Additionally, Single-Cell ProtEomics by MS (SCoPE-MS) and the second-generation SCoPE2 are also technologies that allow the quantification and identification of thousands of proteins through hundreds of single-cell samples with LC-MS/MS [2,5]. They combine a cell lysis protocol compatible with mass-spectrometry and a protein carrier for increasing the sample available for sequencing. The development of multiplexed experimental designs was fundamental, in which carrier proteins from single cells and the total cell lysate are barcoded and mixed [2,40]. By coupling different isobaric tags with different samples, it is possible to investigate how much of a specific protein is present in each sample. Using tandem mass tag reagents, they can distinguish between more than 18 samples in a mixture [3]. This design reduces the loss of proteins from individual cells that adhere to the surfaces of the devices, while improving peptide identification [2,5]. SCoPE-MS has made it possible to classify and investigate the association between mRNA and protein levels, demonstrating that—even in single mammalian cells—covariation of the mRNA is predictive of protein covariation. Based on their proteomes, it also characterizes single-cell gene regulation quantitatively and classifies cell types [40]. SCoPE2 offers a simpler protocol for cell lysis and an optimized pipeline analysis that is widely available and scalable for production use [3]. The sonication is replaced by lysing cells with a freeze–heat cycle in pure water, enabling the quantification—after ten days of instrument time—of over 3042 proteins in 1490 single monocytes and macrophages, thereby discerning single cells by cell type [5,41]. Alternative technological advances have the capability to increase the quantification accuracy and the number of analyzed cells, while allowing the quantification of protein modifications at single-cell level. For example, the carrier protein approach [40] can quantify PTMs with a carrier of peptides avoiding enrichment-associated protein losses. Moreover, MS methods have the potential to measure protein complex formation and composition by crosslinking of the polypeptide chains, and subcellular localization if the complex is closed to organelle-specific proteins. However, such analysis remains to be applied to single-cell MS, and a trade-off still exists between proteome coverage and sample size [1,2].

To provide MS proteomics data quantitatively accurately from single cells and answer biological questions, True Single-Cell Proteomics is also emerging [34]. The workflow couples sample preparation miniaturization and low-flow liquid chromatography with an emerging mass spectrometer, a timsTOF SCP (Bruker, Billerica, MA, USA) that revolutionizes quantitative single-cell biology research with unbiased, deep single-cell 4D-Proteomics™, immunopeptidomics, epiproteomics and analysis of PTMs, complementing scRNA-seq and increasing sensitivity by one order of magnitude [42]. True-SCP based on MS requires no-loss sample preparation by protein isolation and solubilization, followed by tryptic protein digestion and purification of the peptides for MS analysis. It also dissects cell-cycle states due to drug perturbation, as demonstrated by Brunner et al., using HeLa cells. They quantified 1441 proteins per arrested cell in the different stages. The True-SCP dataset covered proteins mapped to different cellular compartments and biological processes, such as metabolism, transport, regulation, or signal transduction with high quantitative accuracy. They also quantified cellular heterogeneity following targeted perturbation to analyze drug responses in single-cell hierarchies on the proteome level [34]. Some other mass spectrometers arising for SCP analysis are Zeno-TOF 7600 (ABSciex, USA) and Orbitrap Exploris 480 (ThermoFisher Scientific, Waltham, MA, USA). Zeno-TOF 7600 is a high-resolution mass system that combines the Zeno trap pulsing with Electron Activated Dissociation (EAD) fragmentation technology to uncover previously inaccessible structural information and detect up to 20× more ions [43]. Orbitrap Exploris 480 allows high-performance, high-throughput insights, combining higher sensitivity and spectral quality for increased productivity, proteome coverage, and maximum certainty in small- and large-scale studies, as well as robust and reliable performance with integrated instrument control, data processing, and servicing software [44].

Furthermore, large-scale single-cell analyses are fundamental to studying biological heterogeneity within complex cell systems such as cancer (since tumors are formed by a multitude of cell types, all functioning in concert), but have been limited to technologies based on RNA and overlooking protein levels [45]. Schoof et al., presented a novel LC–MS-based experimental workflow that enables the study of cellular heterogeneity within a primary leukemia inspired in the initial ScoPE-MS approach. This increased the throughput characterizations and the quantitative accuracy, integrating data from single-cell FACS sorts and a computational workflow for data analysis called Sceptre (single cell proteomics readout of expression). The team quantified about 1000 proteins per cell and analyzed more than a hundred cells per day of instrument time. In addition, they identified heterogeneity and specific proteins in the cell that may represent a starting point for additional research into unknown cell states and potential therapeutic targets [35].

## 4. Software Development

As is well known, thousands of proteins can be identified and quantified by LC-MS/MS in microgram-level samples. However, sometimes—as in the case of mammalian single cell proteomes—they are not enough for a reliable identification of peptides [46]. SCP aims to identify and quantify the complete set of proteins for individual cells in a sample, yet many low-abundance peptides generate few fragment ions—not enough for confident peptide identification—which can impact the features of MS2 fragmentation spectra [46,47]. Since SCP has only recently been developed, there are no algorithms available for single-cell data specifically, and the spectra have different characteristics that might affect the software success and the identification of the best peptide/spectrum matches [47]. Increasing efforts are now focused on improving the data quality, optimizing the sample preparation [48], chromatography, data acquisition and instrumentation [49]. Some important features of current algorithms used to distinguish correct peptide/spectrum matches are i. how many fragment ion peaks match in the theoretical and the observed spectrum, ii. a higher peak intensity of the fragment ion compared with the background, iii. the spectra obtained from the same peptide is expected to be similar [47].

The IceR is a high identification tool that adapts the concept of match-between-runs for both ultra-low inputs and a possible absence in MS1 spectra of isotopic ions, improving the ability to discriminate among single cells [50] (Table 2). Another potential tool is identification algorithms based on tandem mass spectrometry, which identify the most likely peptide candidate by matching the sequence of a certain peptide to an acquired spectrum [47]. In addition, early algorithms for peptide identification, such as SEQUEST [51], relied on matching peaks of fragment ions from the observed spectrum versus the theoretical (Table 2). Next-generation algorithms are able to score individual peaks probabilistically based on predicted intensities and isotopic profiles [47], generating de novo algorithms [52,53] and using them for database search tools [54]. Boekweg et al. characterized variability between single-cell and bulk spectra by examining the three main features on peptide identification performance. All showed single-cell significant changes that potentially impact the success of all peptide identification tools. Among these differences, they indicated an important loss of annotated fragment ions, obtaining fewer peaks in the single-cell spectra than in the bulk spectra, which affected the scoring of peptide/spectrum matches in the algorithms. Additionally, they found that SCP spectra were internally consistent, yet varied from bulk proteomics spectra, suggesting that single-cell spectrum identification and prediction methods should be performed with libraries from single-cell samples instead of bulk libraries [47].

To identify weak-spectrum peptides, Chen et al. developed Data-driven Alignment of Retention Times for IDentification (DART-ID), which uses an ion retention time as well as its spectra for a more confident characterization (Table 2). DART-ID intends to analyze all MS2 spectra, including very-low-confidence peptide/spectrum matches, across experiments as additional evidence for increasing the samples in which proteins are identified and quantified confidently, minimizing assumptions. It also combines them with accurate retention time to update the confidence within the Bayesian framework for global retention time alignment. It increased the number of data points by 30–50% and, thus, decreased the missing data for both bulk and single-cell samples. Evidence from benchmark tests indicated outstanding peptide quantification, upgraded by DART-ID and supporting its utility for quantitative analysis [46]. 

Huffman et al. created Data-driven Optimization of MS (DO-MS), an open-source platform for their interactive visualization and analysis, which diagnoses problems and suggests solutions as specifically as possible (Table 2). To enable targeted diagnostics, the DO-MS dashboard provides juxtaposed distribution plots of data across multiple levels of LC-MS/MS analysis, covering retention lengths at the base and mid-height, all ion and precursor intensity chosen for MS/MS, shift of the elution peak apex, MS/MS event number, identified peptides at all confidence levels, and quantification benchmarks. The use of DO-MS in order to optimize the sampling of the elution peak apexes resulted in increased ion accumulation times and vertex sampling, obtaining a more efficient delivery of ions for MS2 analysis [55].

## 5. Single-Cell Proteomics in Cancer

A complete immune system and tumor microenvironment screening is essential for an accurate and successful cancer therapy. The immune system is complex and requires long-term follow-up of the patient to ensure successful treatments. Understanding intra- and anti-tumor dynamics and heterogeneity is one of the major challenges that can be assisted by proteomic techniques [56]. Specifically, SCP in cancer research has transformed the understanding of the dynamism and biological features of tumors, enabling unbiased analysis of individual malignant cells [57], and, therefore, providing better diagnoses based on their molecular characteristics [58]. It can be used for the identification of i. rare subpopulations of cells; ii. circulating tumor cells; iii. tumor microenvironment characterization; iv. molecular subtyping and tumor heterogeneity; v. progression, tumorigenesis, metastasis, or treatment resistance mechanisms; vi. cancer stem cells [57].

One of the main goals of nanomedicine is to find precise and early indicators of the disease; in particular, anti-tumor immunotherapy has shown great efficacy in earlier stages of the disease [59,60]. For example, chimeric antigen receptor (CAR) therapies and immune-checkpoint blockade therapy have demonstrated that they could trigger specific anti-tumor responses and thus achieve a high rate of complete remission in comparison with other conventional cancer therapies [61,62] (Table 3). In this regard, there is a pressing need for an accurate quality control for CAR products prior to an optimal patient infusion. The study of biomarkers to assess tumor status may be the key to reducing side effects and benefiting more patients [60]. Nevertheless, due to the complex immune response and heterogeneous functionality of immune cells, it continues to be a challenge to identify predictive biomarkers capable of correlating the efficacy and side effects of immunotherapies to develop a more precision medicine [59,60]. To address this issue, several useful approaches have been developed. The single-cell IsoCode chip is a highly multiplexed chip with an antibody barcode array that, combined with ELISA assay and fluorescence signal detection, enables a simultaneous detection up to 40 secreted proteins from individual cells [63], showing a large portion of functions for each immune cell type, and deciphering the functional heterogeneity of responding immune cells among individual patients [60] (Table 3). The IsoCode chip, additionally, measures the Polyfunctional Strength Index (PSI™), a single-cell metric that allows disclosure of in-depth functional heterogeneity not observed previously and predicts clinical response and toxicities of CAR products [60,64,65]. Another example is prostate cancer, which shows multiple genomic alterations and heterogeneity at the proteomic level. Single-cell technologies can capture significant cell-to-cell variability, responsible for heterogeneity within biomarker expression that can be missed when the molecular disturbances are based on bulk tissue samples [66].

Additionally, there are no high-throughput single-cell techniques capable of capturing both changes in phosphorylation levels and gene expression patterns [67,68]. Rivello et al. presented a quantification of RNA and intracellular epitopes by sequencing (QuRIE-seq), a high-throughput platform for quantifying intra- and extracellular (phospho)proteins simultaneously, and the transcriptome within thousands of single cells. This method makes it possible to further understand how biochemical information flows through signaling pathways within the cells upon external stimulation. They applied QuRIE-seq to quantify cell-state changes at signaling and transcriptome level after stimulation of the B-cell receptor pathway in Burkitt lymphoma cells [67] (Table 3).

**Table 3:** Recent works in single-cell proteomics.

Technique	Description	Research	Reference
Single-cell IsoCode chip	Highly multiplexed chip with an antibody barcode array. Simultaneous detection of secreted proteins from individual cells.	They deciphered functional heterogeneity among patients and predicts clinical response and toxicities of CAR products.	Liu, D., et al. [60]
Chimeric Antigen Receptor (CAR)	Single-pass transmembrane receptor to target cancer cells, achieving a high rate of remission. Mainly used against hematological malignancies	CD19-CAR T cell stimulation activated CD19-CAR-specific pathways and canonical TCR signaling.	Griffith, A. A., et al. [61]
Quantification of RNA and intracellular epitopes by sequencing (QuRIE-seq)	High-throughput droplet-based platform to quantify single-cell RNA and (phospho)protein by sequencing within thousands of single cells.	They identified cell-state changes at signaling and transcriptome level after stimulation of the B-cell receptor pathway in Burkitt’s lymphoma cells.	Rivello, F., et al. [67]
Deep Visual Proteomics (DVP)	Image analysis of individual tumor cells based on artificial intelligence combined with single-cell/nucleus laser microdissection and ultra-high-sensitivity MS.	Changes in the proteome on melanocytes progressing to melanoma were characterized, uncovering pathways that spatially vary as the cancer progresses.	Mund, A., et al. [69]
Single Cell ProtEomics by Mass Spectrometry (SCoPE2)	An automated and miniaturized sample preparation workflow to increase quantitative accuracy and throughput while lowering cost and time.	Exploration of monocytes differentiation into macrophage-like cells uncovered a gradient of proteome states in the absence of polarizing cytokines.	Specht, H., et al. [41]
Single-pot Solid-phase enhanced Sample preparation (SP3)	An approach using functionalized paramagnetic beads to trap peptides within a magnetic field, optimizing the sample amount needed while maximizing analyte recovery.	Single-cell analysis of the human oocytes’ proteome identified differential protein expression and fundamental preservation of the genome integrity during maturation.	Virant-Klun, I., et al. [24]

Recently, a novel approach for spatial characterization of single cells called Deep Visual Proteomics (DVP) has been developed [69]. DVP offers an innovative perspective in which sub-micron-resolution imaging analysis of cellular phenotypes based on artificial intelligence is combined with automated laser microdissection. In addition, this technique presents unbiased ultra-high-sensitivity mass spectrometry of single tumor cell; thus, coupling protein abundance and proteome variation with cellular phenotypes while preserving complete spatial information in their native tissue. The software BIAS (Biology Image Analysis Software) makes it possible to coordinate both scanning and laser microdissection microscopes for accurate definition of their morphology, identity, and heterogeneity [69]. Mund et al. identified changes in the proteome over time on melanocytes progressing to melanoma using DVP in a primary melanoma tissue (Table 3). This methodology uncovers pathways that vary spatially as the cancer progresses. For example, dysregulation of mRNA splicing that matches with the reduction of interferon signaling and antigen presentation, as well as the extracellular matrix degradation in metastatic growth [69]. Furthermore, by excising nuclei individually from cell culture, they also classified different cell states based on the proteomic profiles of uncharacterized proteins. Therefore, DVP is able to quantify thousands of proteins in a tumor cell in an unbiased manner, identify tissue or cell-type-specific proteomes, or uncover tumor evolution mechanisms in order to discover therapeutic targets for potential drugs and treatments. The downstream bioinformatic analysis reveals the cell function assigned from the imaging data. Using improved proteomic technologies, DVP will also be adequate for studying single-cell proteoforms or post-translational modifications [69].

## 6. Further Applications

SCP have many applications in biomedical research and nanomedicine. In some cases, they may overlap with single-cell RNA-seq purposes, such as sorting cell states and types, while others may be accomplished only by protein measurement, for example regenerative therapies using rational engineering of directed cell differentiation [2]. However, only some of the cells differentiates into the expected cell type; therefore, such cells may not capture the desired physiological phenotypes completely [70]. Next-generation SCP analysis offers an alternative identifying the signaling processes that lead cell differentiation and simulating them by using agonists or antagonists, which should make it possible to recapitulate it in induced pluripotent stem cells [2]. SCP may also have clinical applications, since protein concentration measurement facilitates the assay development for testing protein degradation-inducing therapies [71], along with identifying the molecular interactions that drive from a genotype or a specific stimulus to a particular phenotype, facing a challenge for proteins and their PTMs. This arises due to the molecules interacting within a pathway, which are rarely measured in a broad range of phenotypic states to constrain models of cellular network [2,6]. For example, the absence of protein measurements in a direct way, which hinder the ability to study signaling networks as the majority of key regulatory parameters are lacking in the data and, therefore, it tends to make some assumptions about specific interactions, reducing the robustness of the results [2,7]. Single-cell protein analysis technologies of the next generation lower those assumptions and enhance the validity of mechanisms deduced, since DNA, RNA, proteins and metabolites are measured across thousands of cells, identifying direct molecular interactions without assumptions about basic aspects of the pathway, understanding the intra- and extracellular regulatory mechanisms [2].

For the application of MS nanotechnologies to the analysis of single cells, the SCoPE-MS approach introduced the idea of using an isobaric carrier, which fulfills three critical functions: i. to minimize loss of sample; ii. to improve ion detectability during MS1 scans; and iii. to provide fragment ions to identify the peptide sequence. Combining this approach with a cell lysis compatible with MS makes it possible to quantify proteins from single cells [41]. Increasing the number of cells and proteoforms quantified reduces the assumptions needed for the analysis [2], so SCoPE2 increases the number of proteins analyzed in a more economical way and, moreover, it enables to make quantitative measurements using enough ion copies per protein [41]. For example, macrophages, according to their polarization, can have pro-inflammatory (M1 polarization) or anti-inflammatory (M2 polarization) functions and be involved in the development and maintenance of tissues [72]. The diversity that exists between these phenotypes cannot be explored at the level of single-cell proteomes due to the limitations of single-cell protein analysis. Nevertheless, the development of SCoPE2 enabled the analysis of monocytes differentiation into macrophage-like cells, uncovering a continuous gradient of proteome states that revealed the possibility of macrophages’ heterogeneity emerging due to the absence of polarizing cytokines (Table 3). It also allowed the research of regulatory networks, such as interactions between p53, its transcript, and the genes regulated by this tumor suppressor [41,72]. In addition, novel methods have been developed to optimize MS data acquisition (DO-MS) [55], as well as for its interpretation once acquired (DART-ID), improving peptide identification and quantification when combined with SCoPE2 [41,46]. Another cell type that undergoes a range of complex processes is mammalian oocytes, through oogenesis, maturation, fertilization, and early embryonic development [24,73]. To understand proteome composition and diversity during maturation, some research has been performed on gene expression programs in human oocytes [74,75,76], yet SCP analysis is needed to characterize functional protein products at different growth and maturation stages [24]. Starting from 100 oocytes, Virant-Klun et al. identified 2154 proteins and more than 300 in their secretome, which located oocytes as largely resting cells with a proteome that was customized for cellular attachment, homeostasis, and environmental interactions via secretory factors. Exploiting the SP3 approach, they scaled down single-cell proteome analysis for human oocytes and identified ∼450 proteins from individual oocytes, showing differential expression of proteins involved in DNA replication which indicated that the preservation of genome integrity is essential during oocyte maturation [24] (Table 3).

Furthermore, some multi-omic technologies are able to evaluate complex molecular, cellular or physiological biomarkers, both quantitative and qualitative, in order to gain a closer understanding of the nature of aging [77] and cardiovascular disease [78]. For example, coronary artery disease is one of the most common causes of cardiovascular death around the world, yet the mechanisms of the implicated genes are not well understood [78]. Recent studies [79,80] have developed and optimized high-throughput technologies for the integration of multi-omics data to identify novel mechanisms and plasma biomarkers, and understand the dynamic interactions involved in these diseases [78].

## 7. Conclusions and Perspectives

Life is derived from dynamic interactions occurring among individual cells and the state of each one of them, as well as the ability to response to environmental signals monitored by protein abundances. Until recently, researchers have been limited to bulk proteomic analyses with samples derived from millions of cells, providing deep protein coverage while eliminating heterogeneity between individual cells [47]. In multicellular organisms, homogeneous model systems are rare, even within isogenic cell populations [2,11]. Thus, single-cell analysis is gaining popularity, using the ability of MS in quantitative protein analysis of single mammalian cells [81,82]. 

**Figure 4:**
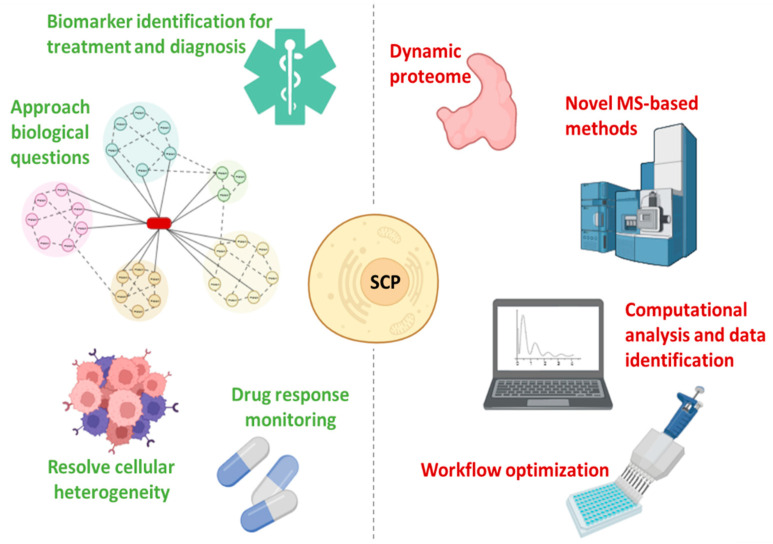
Advantages (green) and challenges (red) in single-cell proteomics.

SCP offers a unique perspective that will drive the further development of single-cell biology since it enables the analysis of hundreds of individual cells per day while quantifying thousands of proteins, allowing the characterization of the functional state of cells [47,82] (Figure 4). Furthermore, the in-depth proteogenomic analysis of individual cells allows the dissection of pathophysiological mechanisms in heterogeneous tissues [6,83]. SCP requires the adoption of MS-based methods, the application of strict quality control standards, and continuous nanotechnology progress, introducing numerous innovations (such as highly parallelized analysis) while increasing throughput, quantitative accuracy, and accessibility [82]. Making SCP affordable also requires computational pathways for the analysis and interpretation of the data, which can be explored by scRNA-Seq software tools [41,82]. Understanding the differences between bulk and single-cell spectra is critical for the optimization of SCP algorithms [47,82] (Figure 4).

To scale up SCP, there are two main requirements: i. make robust and widely available approaches, i.e., accessible, and ii. raise the total number of individual cells analyzed per data project, thus allowing for high throughput to achieve sufficient statistical power [81,82]. The throughput of SCP is determined both by the throughput of sample preparation and MS analysis. Prior to the introduction of automated multi-well plate methods, such as mPOP or nPOP, the limiting step was sample preparation. These have allowed the simultaneous processing of thousands of single cells and the reduction of batch effects, thus causing the rate of sample analysis per MS to become a limiting factor [82]. One of the main approaches for relieving this limitation is increased multiplexing, which can be combined with pooling peptide fragments through single cells, enhancing the identification of different peptide sequences [84]. However, certain batch effects are still present, and computational corrections may be required [82]. The other perspective to overcome the limitation of MS analysis—as shown with bulk samples—is a decreased MS time per sample, which will decrease the pool of peptides to be analyzed by data-dependent acquisition, but potentially supporting high-throughput analysis by data-independent acquisition [85,86]. Further combination of enhanced DIA multiplexing and short separation gradients appears to be the most challenging strategy for high-throughput and high-depth quantitative SCP [82].

Therefore, the emerging need for protein analysis at the single cell level has been a major thrust in MS-based SPC, enabling the development of numerous methods and protocols capable of quantifying more than a thousand proteins from each cell, while analyzing hundreds of single cells. To leverage these capabilities, robust and accessible analytical pipelines and procedures are required, thus driving the progress of further analytical and computational SCP tools (i.e., a set of standards to ensure rigor in the interpretation of data is essential) (Figure 4). The role of nanotechnology has been highly relevant in developing SCP. Furthermore, the next generation of nanotechnology approaches based on proteomics will complement the ongoing methods, while transferring the attention from description to functional characterization of cellular states.

## Figures and Tables

**Table 1:** Main sample preparation methods used in single-cell proteomics.

Sample Preparation Methods	Description	
	Protocol	Advantages
Filter-Aided Sample Preparation (FASP)	Proteins are retained in a filter membrane and are accessible for enzymatic digestion. The generated peptides are collected by centrifugation.	This technique allows the removal of SDS and other low-molecular weight contaminants.
in-StageTip digestion (iST)	Complete sample preparation performed in a StageTip. The final peptide is picked by solid-phase extraction.	It avoids the filter membrane.
Single-Pot Solid-Phase enhanced Sample Preparation (SP3)	It is carried out using functionalized paramagnetic beads to trap peptides within a magnetic field.	Efficient removal of contaminants by washing with different organic solvents.
Minimal ProteOmic sample Preparation (mPOP)	MS compatible digestion buffer which eliminates cleanup steps and minimizes the sample volume used.	It makes it possible to automate sample preparation with PCR thermocyclers, enabling processing many samples in parallel.
Automated nano-ProteOmic sample Preparation (nPOP)	It uses a piezo acoustic device to isolate individual cells using small volumes.	It is a miniaturized and massively parallel method for high throughput.

**Table 2:** Summary of the main computational tools developed in single-cell proteomics.

Software Name	Software Info
Ion current extraction Re-quantification (IceR)	High rates of data-dependent acquisition identification with low missing value rates. More reliably quantified proteins and improved discriminability between single-cell populations.
Peptide identification algorithms (SEQUEST)	Normalize the theoretical spectra by forcing the b-type and y-type ions to be the most intense. It calculates the correlation score (Xcorr) and the ΔCn score.
Data-driven Alignment of Retention Times for IDentification (DART-ID)	Leverage reproducible retention times to increase peptide identifications in LC-MS/MS proteomics. Useful for MS2-based quantification.
Data-driven Optimization of MS (DO-MS)	Diagnose LC-MS/MS problems and enable to rationally optimize them. Data are visualized as full distributions using vertically oriented histograms, allowing subpopulations of ions to be identified.

## Data Availability

Not applicable.
